# Repair of osteonecrosis of the femoral head: 3D printed Cervi cornus Colla deproteinized bone scaffolds

**DOI:** 10.1007/s00132-018-03678-2

**Published:** 2019-01-17

**Authors:** Ping Wang, Gang Li, Wen Qin, Bin Shi, Fan-Jie Liu, Lei-Lei Wang, Bo‑Nian Zhao, Tie-feng Sun, Ling Lin, Dan-Dan Wang

**Affiliations:** 1 State Key Laboratory of Precision Measurement Technology and Instruments, Tianjin University, 300072 Tianjin, China; 2 Shandong Academy of Chinese Medicine, 250014 Jinan, China; 3 Shandong University Hospital, 250100 Jinan, China; 4 Shandong Medicinal Biotechnology Centre, Shandong Academy of Medical Sciences, 250062 Jinan, China; 5 Key Laboratory for Applied Microbiology of Shandong Province, Ecology Institute of Shandong Academy of Sciences, 250014 Jinan, China

**Keywords:** Animal experiments, Traditional Chinese medicine, Morbidity, Polycaprolactone, Osteoblasts, Tierversuche, Traditionelle Chinesische Medizin, Morbidität, Polycaprolacton, Osteoblasten

## Abstract

**Background:**

Osteonecrosis of the femoral head (ONFH) is a common joint disease and a major cause of morbidity.

**Objective:**

In this study Cervi cornus Colla (CCC) deproteinized bone scaffolds were designed and three dimensional (3D)-printed for the repair of ONFH in rats.

**Material and methods:**

The CCC-deproteinized bone scaffolds were 3D-printed using polycaprolactone mixed with the CCC-deproteinized bone powder. The scaffolds were viewed under a scanning electron microscope and subjected to compression analysis. Osteoblasts were isolated from rats and coated onto the scaffolds. Cell proliferation assays were performed with the MTT (3‑[4,5-dimethylthiazole‑2]-2,5-diphenyltetrazolium bromide) kit from Promega. An ONFH was induced in rats and a CCC-deproteinized bone scaffold was implanted into the necrotic femoral head. General observations, X‑ray imaging, and pathological examination of the femoral head were performed to evaluate the treatment of ONFH in the rats.

**Results:**

The scaffolds were porous with a mean pore diameter of 315.70 ± 41.52 nm and a porosity of 72.86 ± 5.45% and exhibited favorable mechanical properties and degradation. In vitro assays showed that osteoblasts accumulated in the pores and adhered to the scaffolds. The CCC-deproteinized bone scaffolds enhanced the proliferation of osteoblasts. The in vivo experiments revealed that the general observation score of rats in the CCC-scaffold implanted group was significantly higher than that in the control group. The X‑ray images showed significant alleviation of ONFH in the CCC-deproteinized bone scaffold implanted rats. The femoral heads of rats in the treatment group showed less destruction or ossification of cartilage cells, few bone cement lines, very little necrosis or irregularities on the cartilage surface and only a small amount of inflammatory cell infiltration in the medullary cavity.

**Conclusion:**

These results suggest that CCC-deproteinized bone scaffold implants facilitated the repair of ONFH in rats. This research provides a new therapeutic approach for the repair of early and mid-term ONFH.

## Zusammenfassung

**Hintergrund:**

Die Osteonekrose des Hüftkopfes (ONFH) ist eine häufige Gelenkerkrankung und eine der wichtigsten Ursachen für Morbidität.

**Ziel:**

In dieser Studie wurden deproteinisierte Knochengerüste aus Hirschhorngelatine (Cervi cornus colla, CCC) entwickelt und mittels eines 3‑D-Druckers hergestellt, um eine ONFH bei Ratten zu behandeln.

**Material und Methoden:**

Die deproteinisierten CCC-Knochengerüste wurden mittels eines 3‑D-Druckers hergestellt unter Verwendung von Polycaprolacton gemischt mit deproteinisiertem CCC-Knochenmehl. Die Gerüste wurden unter einem Rasterelektronenmikroskop betrachtet und einer Kompressionsanalyse unterzogen. Osteoblasten wurden aus Ratten isoliert und auf die Gerüste gezogen. Zellproliferations-Assays wurden mittels des MTT-Kits von Promega durchgeführt. Eine ONFH wurde bei Ratten induziert, und ein deproteinisiertes CCC-Knochengerüst wurde in den nekrotischen Hüftkopf implantiert. Allgemeine Beobachtungen, Röntgenaufnahmen und pathologische Untersuchungen des Hüftkopfes wurden durchgeführt, um die Behandlungen einer ONFH bei Ratten zu evaluieren.

**Ergebnisse:**

Die Gerüste waren porös mit einem mittleren Durchmesser von 315,70 ± 41,52 nm und einer Porosität von 72,86 ± 5,45 %. Sie zeigten günstige mechanische Eigenschaften und Degeneration. In-vitro-Assays zeigten, dass sich Osteoblasten in den Poren ansammelten und an den Gerüsten anhafteten. Die deproteinisierten CCC-Knochengerüste verbesserten die Proliferation der Osteoblasten. Die In-vivo-Experimente zeigten, dass der allgemeine Beobachtungsscore von Ratten in der Gruppe, welcher ein CCC-Knochengerüst implantiert worden war, signifikant höher war als in der Kontrollgruppe. Die Röntgenaufnahmen zeigten eine signifikante Verminderung der ONFH bei Ratten mit einem implantierten CCC-Knochengerüst. Die Hüftköpfe von Ratten in der Behandlungsgruppe zeigten weniger Destruktion oder Ossifikation der Knorpelzellen, wenige Knochenzementlinien und eine sehr geringe Infiltration in der medullären Kavität.

**Schlussfolgerung:**

Diese Ergebnisse zeigen, dass deproteinisierte CCC-Knochengerüstimplantate die Rekonstruktion unterstützen. Diese Studie stellt einen neuen Therapieansatz für die Behandlung einer frühen und mittelfristigen ONFH vor.

## Introduction

Osteonecrosis of the femoral head (ONFH) is a common refractory joint disease [1, 2]. Due to its association with high morbidity and disability, ONFH represents a significant financial burden for patients, families and healthcare systems. Multiple possible causes of ONFH include alcohol abuse, HIV infections and organ transplantation [3, 4]. The prevalent treatment for ONFH is total hip arthroplasty; however, the severe trauma, long recovery period, high medical expense and high risk of side effects associated with this procedure impose a heavy burden on patients, both mentally and physically [5, 6]. As such, less invasive therapies for the treatment of ONFH are becoming increasingly more popular. The use of 3D printing, also known as additive manufacturing (AM), refers to processes used to create three-dimensional objects in which layers of material are formed under automated control to create an object by adding material layer by layer rather than by subtraction from raw material, as is the case with conventional technologies [7]. The 3D objects can be of almost any geometry and are produced using digital model data from a 3D model or another electronic data source, such as an AM file (AMF) [8]. In recent years, 3D printing has become widely known and broadly applied in medical and healthcare fields, such as dentistry, orthopedics and traumatology [8, 9]. It can facilitate the formation of multiple hierarchical tissue and organ-like structures with high precision, such as small or large bone-like scaffolds with high porosity and microstructure [7]. Moreover, this technology makes patient-specific fabrication and clinical customization achievable [10]. The use of 3D bioprinting is one of the most effective approaches to fabricate macroscale bone implants with high mechanical strength and controllable microstructures [10]. With the above in mind, advanced 3D printing technology was combined with materials that are known to be conducive to osteoblast proliferation and differentiation with a view to repairing bone defects or osteonecrosis.

Cervi cornus Colla (CCC) is a refined extract from deer antlers, a highly valued traditional Chinese medicine, which has long been considered beneficial for the prevention and treatment of various diseases, such as acute and chronic arthritis, osteoporosis and fractures, as evidenced in animal models and human clinical trials [11–14]. With respect to its chemical composition, CCC is a protein-polysaccharide complex, which contains 16 amino acids, including glycine, proline and glutamic acid [15]. A previous study revealed that CCC promoted the proliferation and osteogenic differentiation of bone marrow-derived mesenchymal stem cells (BMSC) [16]. In the present study CCC-deproteinized bone scaffolds were designed and 3D printed for the repair of ONFH in rats. This research provides support for a new therapeutic approach for the repair of early and mid-term ONFH.

## Material and methods

### Animals and reagents

Wistar rats weighing 180–220 g (license no. scxk [Lu] 20130009) and 8‑day-old Sprague-Dawley rats weighing 25–35 g (license no. scxk [Lu] 20130009) were purchased from Shandong University Laboratory Animal Center. The rats were housed in an animal chamber maintained at 22 ± 2 ℃ with a relative humidity (RH) of 50 ± 5%. The lighting period was maintained at 12/12 h light and dark cycle. Food manufactured by the Laboratory Animal Center of Shandong Province (Jinan, Shandong, China) and tap water were provided ad libitum. All animal experiments were approved by the Institutional Animal Care and Use Committee of The Shandong Academy of Chinese Medicine. Handling of animals strictly followed the ethical guidelines set forth by the European Community guidelines (EEC Directive from 1986; 86/609/EEC).

Deer antler tablets were purchased from Shandong Baiweitang Chinese Herbal Medicine Drinks Slice Co. (Jinan, Shandong, China). Quality management of the deer antler tablets was performed by the Department of Pharmacognosy, Shandong Academy of Chinese Medicine. Quality control was conducted in accordance with the regulations of the Chinese Pharmacopoeia 2015 edition. Fresh adult pig femurs were purchased from the Inzone supermarket (Jinan, Shandong, China). The reagents used in experiments were as follows: 30% H_2_O_2_, ether, analytical absolute ethanol, 0.25% trypsin, phosphate buffered saline solution (PBS), trypsin-EDTA solution, mixed solution of streptomycin, D‑Hank’s solution, alizarin red indicator (Solarbio, Beijing, China), Dulbecco’s modified Eagle’s medium (DMEM-LG), fetal bovine serum (FBS; Hyclone Laboratories, Logan, UT, USA), BCA kit (Beyotime Institute of Biotechnology, Shanghai, China), retinoic acid (production lot number: HL-20151015, Xi’an Huilin Bio-Tech, Shaanxi, China), and the MTT kit (Promega, Madison, WI, USA).

### Cervi Cornus Colla preparation

Colla Cornus Cervi was prepared according to the 2015 edition of Chinese pharmacopoeia [17]. Deer antler tablets were pulverized and mixed with 4–5 times (w/w) distilled water in a round-bottomed flask. The antler tablets were boiled and the extract was removed every 3 h. The distilled water and antler tablet mixture was boiled until the tablets were soft and could be pinched into powder. The extract was collected and concentrated in vacuo (LGJ-10 laboratory freeze dryer, Beijing Songyuan Huaxing Technology Development, Beijing, China) at 50 ℃ until the water content was 11% (w/w %). The extracts were filtered with double gauze and CCC was obtained after cooling to room temperature. The CCC quality identification and sterilization were conducted as previously described [16].

### CCC-deproteinized bone scaffold design and 3D printing

Cancellous bone was obtained from porcine distal femur chopped into 3 mm × 3 mm × 5 mm pieces and then submerged in normal saline (NS) for 24 h. After rinsing with distilled water, these pieces were put into a glass bottle with 30% H_2_O_2_ solution and incubated in a 37 ℃ water bath for 48 h. Soxhlet’s extraction was used to extract and purify the mixture. The extract was then washed with 75% ethanol and tridistilled water in a dual-frequency ultrasonic cleaner (Ningbo Scientz Biotechnology, Ningbo, China) three times. The deproteinized bone powder was obtained after freeze drying overnight. The CCC powder and the deproteinized bone powder were mixed (1:10, mass/mass) and micronized. The mixture was filtered through a strainer (120 µ mesh) and sterilized. A widely used biodegradable material, polycaprolactone (PCL, Seebio Biotech, Shanghai, China), was mixed with the CCC-deproteinized bone powder and used to print the CCC-deproteinized bone scaffolds. Deproteinized bone scaffolds were similarly printed without CCC. All scaffolds were 3D printed by a fused deposition modelling printer (Qingdao Unique Products Develop Co., Qingdao, China).

### Material properties of the scaffolds

#### Observation of the scaffolds

A total of three CCC-deproteinized bone scaffolds were randomly selected for observation under an inverted microscope (Olympus Corporation, Tokyo, Japan). Subsequently, the scaffolds were coated with gold/palladium and observed under a Zeiss SIGMA 300 high resolution field emission scanning electron microscope (Carl Zeiss, Jena, Germany). The pore size of the scaffolds was determined by image analysis software. The porosity of the scaffolds was determined through the improved liquid displacement method. The scaffold was immersed into ethanol (volume V1) in a graduated test tube for 5 min. After negative pressure degasification, the volume of ethanol (immersed with the scaffold) at this time was V2. The scaffold was carefully removed and the residual ethanol volume was V3. The porosity was calculated according to the formula: (V1–V3)/(V2–V3) × 100%. The experiment was performed in triplicate.

#### Measurement of mechanical properties

A total of three scaffoldswere subjected to compression analysis on a Reger universal compression tester (Shenzhen Reger Instrument, Shenzhen, China). Vertical compression tests were used to detect the compression force of the scaffolds. All compression tests were conducted under standard environmental conditions (20 ± 1 ℃, relative humidity, RH 65 ± 2%).

#### Degradation rate determination

A total of three scaffolds were randomly selected and weighed (M1). The scaffolds were submerged in 20 ml PBS and incubated in a thermostat (Jintan Medical Equipment Factory, Jiangsu, China) at 37 ℃ for 6 weeks. The PBS was replaced every week and the mixture was filtered. The residue was collected and weighed (M2). The weight of the scaffolds (M2–M1) was recorded weekly.

### Osteoblast culture and characterization

The 8‑day-old Wistar rats were killed by cervical dislocation and the skull was harvested. The skull bone was placed in a Petri dish with PBS. The endosteum, pericranium and interosseous tissues were removed. After three washes in PBS, the skull bone was cut into small blocks f 1 mm × 1 mm in size. The bone blocks were digested with 0.25% trypsin and type II collagenase for 10 min. After discarding the supernatant, the bone blocks were digested with 5 ml type II collagenase for 30 min. After digestion cells were harvested through a cell strainer (100 µ mesh) and centrifuged (1000 rpm, 5 min). Cells were placed in a 25 cm^2^ flask and cultured in DMEM-LG supplemented with 20% FBS in a cell culture incubator (Changsha Hua Xi Electronics Technetronic, Changsha, China) at 37 ℃ with 5% CO_2_. Culture medium was replaced every 3 days and 8–10 days later, cells were digested with 0.25% trypsin and subcultured.

The third-generation cells were collected and identified with alizarin red staining. After two washes with PBS, osteoblasts were fixed with 95% ethanol for 10 min. After three washes with distilled water, the osteoblasts were stained with 0.1% alizarin red-Tris-HCl (pH 8.3) solution at 37 ℃ for 30 min.

### Proliferation assay

The adherent osteoblasts were digested with 0.25% trypsin and prepared for single cell suspension to a final concentration of 2 × 10^4^ cells/ml. After soaking the scaffold in cultured medium, the scaffold was immersed in 1 ml cell suspension (2 × 10^4^ cells) in a 24-well plate and cultured in an incubator at 37 ℃ with 5% CO_2_. An MTT assay kit was used to evaluate the growth rate and relative cell activity, according to the manufacturer’s protocol. After incubation for 24 h, 48 h, and 72 h, 20 µl MTT was added to each well and incubated for an additional 4 h. The absorbance at 490 nm was measured with a microplate reader (Thermo Fisher Lab system, Waltham, MA, USA). Each experiment was performed in triplicate.

### Repair of ONFH by CCC-deproteinized bone scaffold implanting

After 1 week of acclimatization, 60 Wistar rats weighing 180–220 g were randomly distributed into 3 groups: the control group, the ONFH model group and the CCC-deproteinized bone scaffold implanted group. The rats were given 0.14 g/kg retinoic acid by oral gavage once per day to induce ONFH. After 8 weeks, 2 rats were selected randomly and killed by cervical dislocation. The bone tissue of the femoral head was collected to assess whether treatment with retinoic acid successfully induced ONFH. The rats in the control group served as healthy comparative animals and were not induced ONFH.

The ONFH rats were anesthetized through intraperitoneal injection of 0.3% pentobarbital sodium (1 ml/100g). The rats were fixed in the prone position on the operating table and the right lower limbs were abducted 20°. The CCC-deproteinized bone scaffold was implanted into the necrotic femoral head. Under sterile conditions muscles were separated and the femoral head ligament was exposed. The ligament was slowly peeled off to expose the femoral head. The scaffold was placed to ring the femoral head and fixed and then the muscles were sutured. The necrotic femoral head of the left lower extremity did not receive any treatment. After 10 weeks, general observation, X‑ray imaging and pathological examination of the femoral head were performed. The general observations and score were conducted according to Table 1.

**Table 1:** Scoring standard of general condition of the femoral head in the rats

Investigation items	Index	Specific performance	Score
Behavioral function	Limp	Nothing	7
		Mild	5
		Moderate	3
		Severe	0
Joint mobility	Flexion	>70°	9
		>60°	5
		>30°	2
		<30°	0
	Abduction	>30°	4
		>15°	2
		>5°	1
		<5°	0
	Internal rotation	>15°	2
		>5°	1
		<5°	0
	External rotation	>15°	2
		>5°	1
		<5°	0

### Hematoxylin and eosin (HE) staining and analysis

The femoral heads were fixed in 10% formaldehyde for 24h before proceeding to paraffin embedding. Serial 5‑µ longitudinal sections were stained with hematoxylin and eosin for histopathological examination. The tissue sections were deparaffinized with dimethylbenzene and rehydrated with a 100%, 95%, 90%, 80% and 70% series in ethanol. The sections were stained with hematoxylin for 15 min. After 3 washes with distilled water, the sections were differentiated with 0.3% acid alcohol for 30 s and rinsed under running tap water for 10 min. The sections were stained with eosin for 2 min and rinsed under tap water. Finally, the sections were dehydrated with graded ethanol, cleared and evaluated.

According to Mankin’s scoring principles [18] the osteonecrosis of femoral head score was calculated as the sum of cartilage structure score and the osteocyte necrosis score. Briefly, the cartilage structure was scored as 0 (normal), 1 (disorder of tissues, and clear structure layers), 2 (disorder of structure layers), and 3 (severe disorder of tissues and structure layers). Osteocyte necrosis was scored as 0 (normal), 1 (hypertrophy and hyperplasia of osteocytes), 2 (deep staining of osteocytes nucleus), 3 (pyknosis of osteocytes and disappearance of nucleus).

### Statistical analysis

All experiments were performed in triplicate. All statistical analyses were performed using the Statistical Package for the Social Sciences, version 17.0 (SPSS, Chicago, IL, USA). Comparisons between groups were analyzed using the least significant difference (LSD) test. Quantitative data are reported as mean ± standard deviation. A two-sided *p*-value of less than 0.05 was considered statistically significant.

## Results

### 3D printing of CCC-deproteinized bone scaffolds and deproteinized bone scaffolds

#### Macroscopic and microscopic scaffold observations

**Fig. 1:**
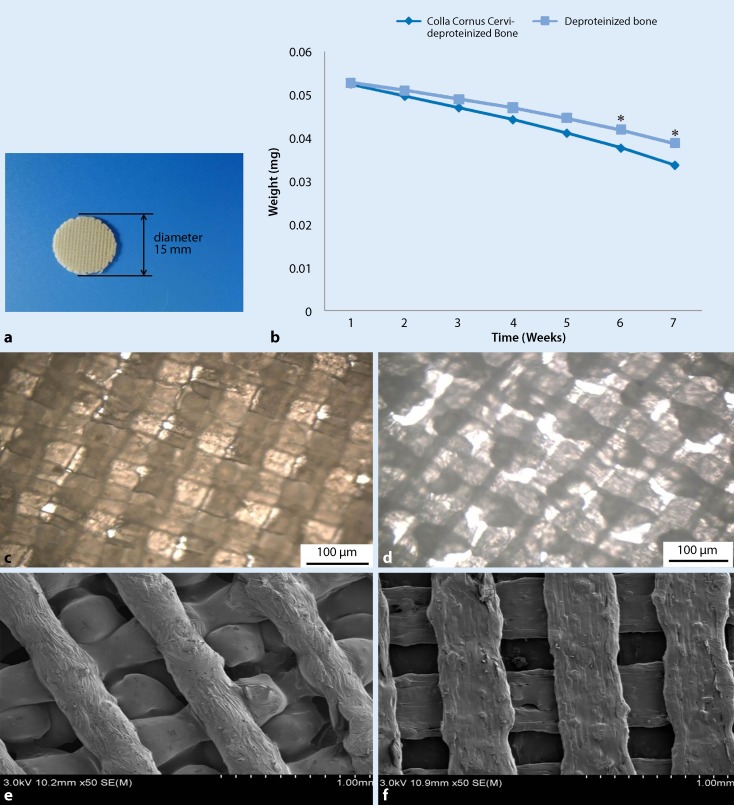
Scaffold characteristics. **a** Exterior view of a 3D printed CCC-deproteinized bone scaffold (with a diameter of 15 mm and a thickness of 3.5 mm). **b** Scaffold degradation curves. The degradation levels of the CCC-deproteinized bone scaffolds and deproteinized bone scaffolds after 6 weeks immersion in PBS reached 35.81% and 26.61%, respectively (*P < 0.05). **c** CCC-deproteinized bone scaffold observed under microscopy (200× magnification). **d** Deproteinized bone scaffold observed under microscopy (200× magnification). **e** CCC-deproteinized bone scaffold observed under SEM. **f** Deproteinized bone scaffolds observed under SEM. *CCC* Cervi cornus Colla, *PBS* phosphate-buffered saline, *SEM* scanning electron microscope

The 3D printed scaffolds with a diameter of 15 mm and a thickness of 3.5 mm were cylindrical, porous, beige in color and had no distinctive odour (Fig. 1a). There were a large number of regular pores, as observed under light microscopy (Fig. 1c, d). The pore structure was smooth and the pores appeared uniform under SEM (Fig. 1e, f). The average pore diameter and porosity of CCC-deproteinized bone scaffolds and deproteinized bone scaffolds are shown in Table 2. The porosity of CCC-deproteinized bone scaffolds (72.86 ± 5.45%) is significantly higher than the deproteinized scaffolds (64.80 ± 3.87%).

**Table 2:** Comparison between CCC-deproteinized bone scaffolds and deproteinized bone scaffolds

Group	Pore diameter (nm)	Porosity (%)	Sample area (nm^2^)	Maximum load (*N*)	Compressive strength (MPa)
CCC-deproteinized bone scaffolds	315.70 ± 41.52	72.86 ± 5.45	9.71 ± 0.45	283.41 ± 14.97	6.27 ± 0.96
Deproteinized bone scaffolds	461.30 ± 25.18	64.80 ± 3.87	9.06 ± 0.56	190.57 ± 15.42	4.45 ± 1.02

*CCC* Cervi cornus Colla

#### Mechanical properties

Compression tests revealed that with increasing load, the CCC-deproteinized bone scaffolds and deproteinized bone scaffolds were highly compressed. The deproteinized bone scaffolds broke as the compressive load reached 4.45 ± 1.02 MPa (Table 2). The CCC-deproteinized bone scaffolds broke as the compressive load reached 6.27 ± 0.96 MPa (Table 2). There was no significant difference in compression properties between the two scaffolds.

#### Scaffold degradation

The degradation percentages of the CCC-deproteinized bone scaffolds and deproteinized bone scaffolds after 6 weeks of immersion in PBS were 35.81% and 26.61%, respectively (Fig. 1b; *P* < 0.05). The degradation curves are shown in Fig. 1b.

### Influence of CCC-deproteinized bone scaffolds on the proliferation of osteoblasts

**Fig. 2:**
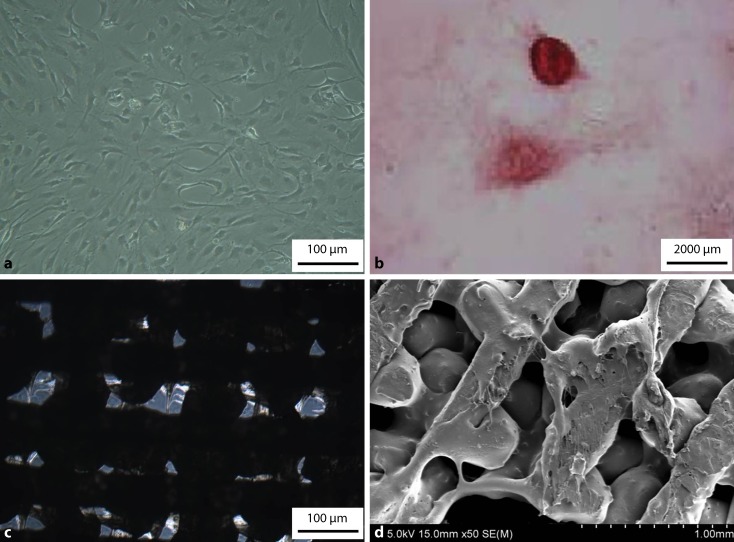
CCC-deproteinized bone scaffolds loaded with osteoblasts. **a** Osteoblasts cultured in vitro (200× magnification). **b** Calcium nodules of the third-generation osteoblasts stained by alizarin red (10× magnification). **c** Osteoblasts cultivated for 72 h on the CCC-deproteinized bone scaffolds imaged under light microscopy (200× magnification). **d** Osteoblasts cultivated for 72 h on the CCC-deproteinized bone scaffolds imaged under SEM. The cells accumulated in the pores and adhered to the scaffolds. The surfaces of the scaffolds were covered with gelatinous extracellular matrix.* CCC* Cervi cornus Colla, *SEM* scanning electron microscope

The results of osteoblast culture and alizarin red staining are detailed in Fig. 2a, b. The SEM images revealed that cells accumulated in the pores and adhered to the scaffolds (Fig. 2c, d). The surface of the scaffold in Fig. 2d can be seen covered with gelatinous extracellular matrix. The MTT assays revealed that the proliferation rate of cells cultivated on CCC-deproteinized bone scaffolds was significantly greater than that of the control group (Table 3). These results suggested that CCC-deproteinized bone scaffolds enhanced the proliferation of osteoblasts.

**Table 3:** Effect of CCC-deproteinized bone scaffolds on the proliferation of osteoblasts (X̅ ± s, *n* = 10)

Incubation time (h)	OD 490 nm	
	Control group	CCC-deproteinized bone scaffolds group
24	0.247 ± 0.016	0.492 ± 0.091^**^
48	0.535 ± 0.093	0.767 ± 0.118^**^
72	0.564 ± 0.047	0.612 ± 0.131^*^

*CCC* Cervi cornus Colla, *OD* Optical density

^*^P < 0.05, ^**^P < 0.01, compared with the control group

### Repair of ONFH using CCC-deproteinized bone scaffolds implanted in rats

#### General observations

**Fig. 3:**
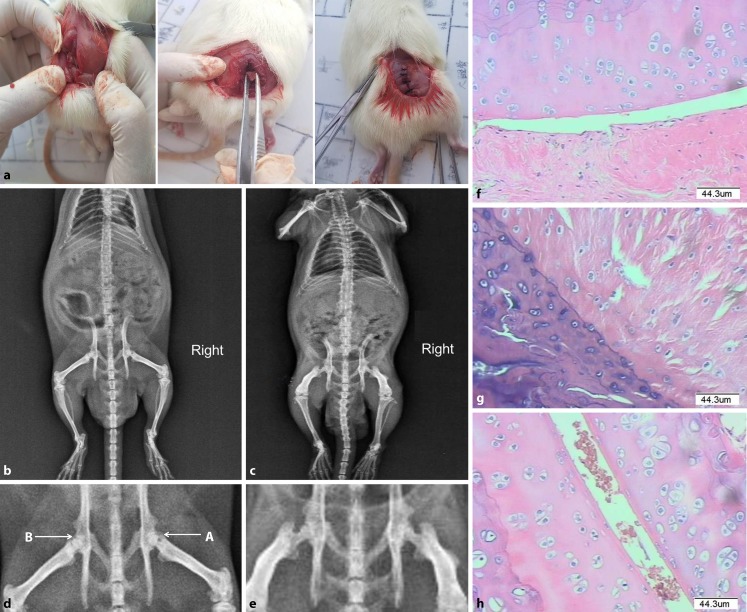
Implanted CCC-deproteinized bone scaffolds promoted repair of ONFH in rats. **a** Operated femoral head of rats showed the operation and scaffold implanted position. **b** X-ray image of a prostrate rat implanted with a CCC-deproteinized bone scaffold in the right lower extremity. **c** X-ray image of an ONFH model rat. **d** Corresponding femoral head of the rat implanted with CCC-deproteinized bone scaffold in **b (***A* the femoral head implanted with a CCC-deproteinized bone scaffold exhibited a smooth articular surface with restored necrotic and cystic areas. The scaffold is integrated and appears blurred with the surrounding tissues, *B* the non-implanted femoral head exhibited cystic degeneration, bone fragments, and articular surface collapse). **e** Corresponding femoral head of the ONFH model rat in **c**. The necrotic femoral head showed cystic degeneration, osteosclerosis, segmental femoral head flattening and subchondral collapse. **f** Pathological observation of the femoral head of a rat in the control group (200× magnification). The detailed image reveals normal ultrastructure with regularly arranged cartilage cells, regular bone cement lines, and no degeneration or necrosis of the cartilage surface. **g** Pathological observation of the femoral head of an ONFH model rat. Results showed osteocyte degeneration and necrosis, ossification of the cartilage, sclerosis of the ligament fibers, and inflammatory cell infiltration in the medullary cavity. **h** Pathological observation of the femoral head of a rat implanted with a CCC-deproteinized bone scaffold. Less destruction or ossification of cartilage cells, few bone cement lines, very few irregular or necrotic cartilage surface areas, with a small quantity of inflammatory cell infiltration observed in the medullary cavity. *CCC* Cervi cornus Colla, *ONFH* Osteonecrosis of the femoral head

The operation and scaffold implanted position in the femoral heads of rats are shown in Fig. 3a. The rats in the model group showed festering in the tails, limping, and limited joint activity. The rats with implanted CCC-deproteinized bone scaffolds showed no obvious change of femoral head shape, no occurrence of incision infections, and no obvious immunological rejection. The leg activity gradually returned to normal, and none of the rats died. The general observation results were recorded and scored and are detailed in Table 4. The results showed that the score of the CCC-deproteinized bone scaffold group was significantly higher than that of the model group (Table 4, *P* < 0.001).

**Table 4:** The general observation score of CCC-deproteinized bone scaffolds in the repair of ONFH in rats (X̅ ± s, *n* = 10)

Group	Score
Control group	23.2 ± 1.32
Model group	4.7 ± 1.16
CCC-deproteinized bone scaffolds implanted group	13.1 ± 1.79^***^

*CCC* Cervi cornus Colla, *ONFH* Osteonecrosis of the femoral head

^***^P < 0.001, compared with the model group

#### X-ray analysis

The necrotic femoral heads of the ONFH rats showed cystic degeneration and osteosclerosis, and some animals displayed segmental femoral head flattening or subchondral collapse (Fig. 3c, e). Significant alleviation of femoral head necrosis was observed in the rats implanted with CCC-deproteinized bone scaffolds (Fig. 3b, d). The X‑ray analysis revealed smooth articular surfaces and restoration of necrotic and cystic areas (Fig. 3d, A), with some rats displaying mild variability in the femoral head, such as mild blurred bone trabecula.

#### Histopathological analysis

In the control group, femoral heads displayed normal ultrastructure, with regularly arranged cartilage cells, regular bone cement lines and no degeneration or necrosis of the cartilage surface (Fig. 3f). In the model group, femoral heads exhibited osteocyte degeneration and necrosis, ossification of the cartilage, sclerosis of the ligament fibers, and inflammatory cell infiltration into the medullary cavity (Fig. 3g). In the implanted group, femoral heads exhibited less destruction or ossification of the cartilage cells, few bone cement lines, very few irregular or necrotic cartilage surface regions, and only a few inflammatory cells infiltrated the medullary cavity (Fig. 3h). The results of histological score and analysis are shown in Table 5. The histological score of CCC-deproteinized bone scaffolds implanted group (4.5 ± 0.81) was significantly lower than that of model group (5.5 ± 0.5, *P* < 0.01). These results suggest that CCC-deproteinized bone scaffold implantation facilitated the repair of ONFH in rats.

**Table 5:** Histological score and analysis of CCC-deproteinized bone scaffolds in the repair of ONFH in rats (X̅ ± s, *n* = 10)

Group	Score
Control group	0
Model group	5.5 ± 0.5
CCC—deproteinized bone scaffolds implanted group	4.5 ± 0.81**

*CCC* Cervi cornus Colla

^**^P < 0.01, compared with the model group

## Discussion

The ONFH is a pathologic process which causes bone cells to die and the femoral head to collapse through the interruption of the blood supply to the femoral bone [4]. Vascularization of large-scale artificial bone tissue grafts is the most significant and critical challenge in the reconstruction of bone defects [19]. A specific approach used to meet this challenge in the treatment of ONFH is to form a model of the collapsed femoral head area under the control of a computer imaging system, to construct a precise 3D structure, before filling and repairing the area of ONFH [8]. During such a reconstructive process, the physical and mechanical properties are evaluated to provide experimental support in clinic aspects [9, 10]. The patient-specific model plays an important role in choosing the most effective approach and strategy [10]. Since 3D printing has become a vital tool in tissue engineering and medicine, there is an ever-growing need to develop new biomaterials that can be 3D printed and can emulate the compositional, structural, and functional complexities of human tissues and organs [10].

There are very few reports about 3D printed scaffolds for the treatment of ONFH. Zhu et al. created a gelatine scaffold embedded in uniquely shaped 3D printed porous titanium parts to treat ONFH in rats. After hybridization with platelets, the scaffold exhibited a low yet considerable rate of stable, and long-term growth factor release [20]. Lv et al. reported a novel 3D printed device for the localization and extraction of trabeculae from human femoral heads, providing a method for researching musculoskeletal degenerative diseases and possibly a better clinical understanding of these disorders [21].

In the present study CCC-deproteinized bone scaffolds were designed and 3D printed with a view to repairing ONFH in rats. A widely applied biodegradable material, PCL, was used to build this scaffold due to its appropriate support strength and favorable cytocompatibility [19]. The CCC-deproteinized bone scaffold was 3D printed with a mixture of CCC powder, deproteinized bone powder, and PCL. The CCC, a refined extract from deer antler, is thought to have bone-invigorating therapeutic effects [22]. Indeed, CCC has been widely used to nourish kidneys and for bone tonifying in traditional Chinese medicine, such as in the treatment of arthritis, osteoporosis [22], hypercholesterolemia [23], and to heal chronic wounds. The CCC contains mineral elements (Ca, Zn, and Pb), carbohydrates, polypeptides, proteins, and some special cell growth factors [24–27]. Previous studies revealed that CCC promoted osteoblast proliferation and enhanced osteoblastic differentiation [16]. To take advantage of these favorable properties, in the present study CCC was incorporated into PCL-based 3D printed scaffolds with a view to promoting osteoblast growth and ONFH repair. Moreover, the bone scaffolds were deproteinized to minimize immunological rejection.

The 3D printed CCC-deproteinized bone scaffolds were porous with a pore diameter of 315.70 ± 41.52 nm and a porosity of 72.86 ± 5.45%, which facilitated good nutrient permeability and good support for the adhesion and proliferation of osteoblasts, as evidenced by the MTT assay. Compression tests revealed that the scaffolds exhibited favorable mechanical properties. As such, the scaffolds should play a favorable support function for long-term in vivo engraftment. Furthermore, the degradation of CCC-deproteinized bone scaffolds and deproteinized bone scaffolds was 35.81% and 26.61% after 6 weeks immersion in PBS, respectively. Microscopical observation revealed that osteoblasts accumulated in the pores and adhered to the scaffolds. Moreover, the scaffold surfaces were covered with gelatinous extracellular matrix. These results indicated that the 3D printed scaffolds were conducive to the expansion of osteoblast numbers. In vitro MTT assays revealed that the proliferation of cells cultivated on CCC-deproteinized bone scaffolds was significantly greater than that of the control group. These results suggest that the incorporation of CCC enhanced the proliferation of osteoblasts.

The CCC-deproteinized bone scaffolds were implanted into the necrotic area of femoral heads to repair ONFH in rats. General observations revealed that rats implanted with CCC-deproteinized bone scaffolds exhibited no obvious change of femoral head shape, no incision infection, and no obvious immunological rejection issues. The general observation score of the implanted group was significantly higher than that of the model group. The X‑ray examinations revealed significant alleviation of femoral head necrosis in the rats implanted with CCC-deproteinized bone scaffolds. Histopathological analysis revealed that the femoral heads in the implanted rats showed little destruction or ossification of cartilage cells, few bone cement lines, very few irregularities or necrotic areas on the cartilage surface, with only a small quantity of inflammatory cells infiltrating the medullary cavity. These in vivo results suggest that CCC-deproteinized bone scaffold implants facilitated the repair of ONFH in rats.

For the mechanism study it was reported that angiogenesis provides nutritional support to osteoblast growth and has previously been shown to be a key factor influencing ingrowth of epithelium [28, 29]. Wang et al. revealed that extract of CCC bone promoted the expression of vascular endothelial growth factor [30]. Previous studies suggested that antler extract had a positive curative effect on avascular necrosis of the femoral head in rats [31]. In the present study, CCC-deproteinized bone scaffold implants facilitated the repair of osteonecrosis. Based on these findings, vascularization may play important roles in the repair of osteonecrosis. In further studies, the effect of CCC-deproteinized bone scaffold on vascularization and molecular mechanisms will be investigated to evaluate the repair of ONFH in rats.

## Conclusion

In the present study CCC-deproteinized bone scaffolds were designed and 3D printed for the repair of ONFH in rats. The scaffolds were porous and exhibited favorable mechanical properties and degradation. In vitro assays showed that CCC-deproteinized bone scaffolds enhanced the proliferation of osteoblasts. In vivo assays with ONFH rats suggested that CCC-deproteinized bone scaffold implants facilitated the repair of ONFH. This research provides a new therapeutic approach for the repair of early and midterm ONFH.

## Abbreviations

AM: Additive manufacturing
BMSC: Bone marrow-derived mesenchymal stem cells
CCC: Cervi cornus Colla
DMEM-LG: Dulbecco’s Modified Eagle’s Medium, low glucose
HIV: Human immunodeficiency virus
LSD: Least significant difference test
ONFH: Osteonecrosis of the femoral head
PBS: Phosphate-buffered saline
RH: Relative humidity
SEM: Scanning electron microscope
